# Protein structure–function relationships as the molecular basis of phenotypic diversity in *GDAP1*-associated Charcot–Marie–Tooth disease

**DOI:** 10.3389/fmed.2026.1965725

**Published:** 2026-10-14

**Authors:** Victoria I. Bunik, Alina A. Emelyanova

**Affiliations:** 1 Belozersky Institute of Physicochemical Biology, Lomonosov Moscow State University, Moscow, Russia; 2 Faculty of Bioengineering and Bioinformatics, Lomonosov Moscow State University, Moscow, Russia

**Keywords:** Charcot–Marie–Tooth disease, clinical phenotype, GDAP1 gene missense mutation, GDAP1 protein variant, half-of-the-sites reactivity, molecular mechanism of CMT

## Abstract

Mutations in the *GDAP1* gene, which encodes the ganglioside-induced differentiation-associated protein 1 (GDAP1)—a member of the detoxifying glutathione S-transferase (GST) superfamily—cause Charcot­­–Marie–Tooth disease (CMT) a genetically heterogeneous group of inherited neuropathies. This mini review correlates the clinical phenotypes of CMT with the molecular dysfunctions of GDAP1. Implicated in mitochondrial dynamics, calcium homeostasis, cellular organelle contacts, and the metabolism of mitochondria, lysosomes, and peroxisomes, the known molecular functions of GDAP1 include localization to mitochondrial outer membrane, dimerization, GST activity, and binding of hydrophobic compounds and proteins. Our analysis reveals that mutation-induced perturbations in specific molecular functions correlate with clinical phenotypes better than mutation class (missense vs. nonsense or frameshift), mode of inheritance (dominant vs. recessive), or mitochondrial morphology. The G327D and truncated GDAP1 variants have similar clinical phenotypes because all lack membrane binding. Early disease onset and rapid progression are observed not only with recessive variants (P111, N227, L239, and R282) but also with dominant variants (C240), if the substituted residues are proximal to the hydrophobic ligand. While dominant GDAP1 variants cause axonal CMT, all the recessive variants inferred to affect the hydrophobic ligand binding present with demyelination in addition to axonal damage. These different disease trajectories may rely on the potential formation of fully functional wild-type GDAP1 homodimers in dominant heterozygotes but not in biallelic recessive cases. Thus, elucidating how disease-associated protein variants affect GDAP1 molecular functions advances our understanding of the molecular basis of clinical phenotypes, which is required for tailored therapies.

## Introduction

Charcot–Marie–Tooth disease (CMT) comprises a group of frequently occurring (1:2,500) inherited neuropathies, presented as demyelinating, axonal, mixed (intermediary), and X-linked major forms. With the increasing availability of genetic testing, the number of CMT-associated genes and their variants is permanently growing. Currently, the OMIM Phenotypic Series PS118220 lists 58 genes (accessed 26 July 2026) associated with formally classified CMT subtypes (1), whereas the broader GeneReviews classification (2) includes 78 genes (accessed 26 July 2026) associated with CMT and overlapping inherited neuropathies. The more extensive ClinVar database covers a broad range of HGVS-described (3) germline and somatic variants, aggregating clinical interpretations as reported by submitters (4). However, incomplete patient-level genotype and zygosity data limit ClinVar’s utility for precise genotype–phenotype analysis, underscoring the need to consult original reports alongside complementary resources such as OMIM (1), GeneReviews (2), and LOVD (5).

The *GDAP1* gene, encoding the ganglioside-induced differentiation-associated protein 1 (GDAP1), is a causal gene for CMT. The curated Global Variome shared LOVD database currently lists 105 GDAP1 variants (accessed 26 July 2026) associated with the CMT subtypes CMT2K, CMT4A, CMTRIA, and CMT with vocal cord paresis (5). Several GDAP1 mutations are associated with a founder effect in specific populations. These mutations include A247V in Japan (6), R120G in the Mediterranean region (7), L239F in Europe (8), Q163X in Spain (9), and H123R in Finland (10). *GDAP1*-associated CMT can exhibit autosomal-dominant (AD) or autosomal-recessive (AR) inheritance.

To advance the understanding of genotype–phenotype relationships, this work analyzes clinical phenotypes in relation to protein and cellular dysfunctions associated with *GDAP1* mutations, with a particular emphasis on missense mutations that permit the synthesis of GDAP1 variants. Throughout this analysis, *GDAP1* mutations are reported relative to the reference *GDAP1* coding and protein sequences (NM_018972.4/NP_061845.2). The analysis includes CMT-associated *GDAP1* missense variants listed in ClinVar (accessed 11 September 2026) for which clinical phenotypes and zygosity are unambiguously documented in published case reports. Available model studies characterizing the molecular alterations in the protein variants and the cellular consequences of their expression are considered to elucidate the molecular mechanisms underlying the clinical phenotypes.

## Genotype–phenotype relationships in *GDAP1* mutations: genetic and protein science views

Attempts have been made to understand the genotype–phenotype relationships in CMT by considering genes as functional units, that is, without differentiation of site-specificity of the gene mutations. In these studies, the aggregated clinical phenotypes of CMT patients carrying variants in one causal gene are compared with those of patients carrying variants in another causal gene (11, 12). However, these approaches have low resolution, as different genes may contribute to the same clinically diagnosed CMT types, while different mutations in one gene may cause different types of the disease, as exemplified for *GDAP1* in Table 1.

**Table 1:** Clinical phenotypes and known molecular dysfunctions caused by CMT-associated missense mutations in *GDAP1* gene.

GDAP1 variant	GDAP1 region	Germline classification	Genetics and clinical phenotype	Molecular and cellular dysfunction studies
Heterozygotes				
S34C (c.101C>G)	N-term GST	Uncertain significance	AD CMT2K Onset at 6–10 years of age Intrafamiliar variability in the onset and severity (43)	
R120W (c.358C>T)	C-term GST	Pathogenic	AD CMT2K Onset ranges between the first and third decade (7, 13, 14, 44). Vocal cord paresis, burning sensation in the feet, intermittent numbness of the hands, reduced pain sensation up to the mid-calf (7, 45). Frequent falling (6). Some patients at about 40 years of age have CMT neuropathy score 0, hypoesthesia in toes, absent ankle jerk or reflexes in lower limbs; the most common variant of AD CMT due to GDAP1 mutations in Spain (13)	In wild-type (WT) GDAP1 R120 interacts with C240, the R120 interaction with R226 is involved in the system of salt bridges. Human R120W GDAP1 exhibits decreased thermal stability, shift in *α*3 by ∼1 Å, perturbed interaction of C240, perturbed salt bridges E222-R226-E229, crystallizes in a different form, compared to WT GDAP1. The variant does not induce morphological changes or toxicity in dorsal root ganglion neurons or HEK293T, does not change cellular localization. Both WT and R120W GDAP1 expressed in the cells do not form intersubunit disulfide bond observed in truncated GDAP1 forms studied *in vitro* (20)
R120G (c.358C>G)	C-term GST	Uncertain significance	AD CMT2K Onset in the first decade, slow progression (7)	
H123R (c.368A>G)	C-term GST	Pathogenic/likely pathogenic	AD CMT2K Onset ranges between the first and third decade Slow progression, variable phenotypes, but all patients remained ambulant; loss of sensation in limb extremities (7, 46) H123R GDAP1 causes up to 14% of CMT2 in Finland (10)	In H123R, GDAP1 π-stacking between H123 and Y124 is lost, intramolecular interaction network is changed, thermal stability decreases. WT and H123R GDAP1 do not differ in their cellular localization. Expression in neuroblastoma cell line SH-SY5Y increases SOCE^a^. Human GDAP1 H123R does not induce morphological changes or toxicity in dorsal root ganglion neurons, HEK293T, and human skin fibroblasts. Both WT and H123R GDAP1 expressed in the cells do not form intersubunit disulfide bond observed upon recombinant expression of truncated GDAP1 forms (20, 47)
E126K (c.376G>A)	C-term GST	Conflicting pathogenicity	AD CMT2 Onset at 15 years of age, ambulant at 52. Poor running (6)	
A156G (c.467C>G)	*α*-Loop	Pathogenic	AD CMT2K Onset ranges between the first and second decade Slow progression, all patients remained ambulant (48)	HeLa cells with this GDAP1 variant have impaired mitochondrial fusion, exhibit mitochondrial fragmentation, and increased sensitivity to apoptosis (48).
T157P (c.469A>C)	*α*-Loop	Pathogenic	AD CMT2K Onset ranges between the second and third decade, with a mean age of 24 years (49). Absence of deep tendon reflexes, optic nerve atrophy (44, 50)	T157P GDAP1 has destabilized *α*-loop and decreased affinity to 4-hydroxynonenal, end-product of lipid peroxidation. In neuroblastoma cell line SH-SY5Y with T157P GDAP1 SOCE increases, mitochondria aggregate in perinuclear zone (30, 47)
Q218E (c.652C>G)	C-term GST	Conflicting pathogenicity	AD CMT2K Onset ranges from the first decade of life to 25 years of age (7, 51). Muscle atrophy of the hands, reduced sensation in the upper and lower limbs, normal or slightly reduced nerve conduction velocity (7, 14, 52)	
R226S (c.678A>C/c.678A>T)	C-term GST	Uncertain significance	AD CMT2K Onset at 8–50 years of age, variable phenotypes, distal paresthesia and dysesthesia. A patient had Holmes-Adie syndrome (7, 43)	
**C240Y (c.719G>A)**	C-term GST	Conflicting pathogenicity	AD CMT2K Onset in the first decade, rapid disease progression, compared to R120W (11, 15, 53)	The activity of the mitochondrial respiratory chain complex I in fibroblasts from patients with C240Y GDAP1 is 40% lower than in the control group, while the diameter of tubular mitochondria is 33% larger and the mitochondrial mass is 20% higher. Mitochondrial biogenesis is increased by 20%. Oxidative stress is increased in fibroblasts of a patient carrying C240Y GDAP1, but not in another patient, whose fibroblasts express R120W GDAP1 (15, 53)
P274L (c.821C>T)	C-term GST	Conflicting pathogenicity	AD CMT2K Onset at 47 years of age, followed by a rapid disease progression and loss of ambulation at 61 years of age. Intrafamiliar variability in clinical penetrance (48)	
A312T (c.934G>A)	HD	Uncertain significance	AD CMT2K Onset ranges from 6 to 7 to 37 years of age. Weakness of arms and legs, steppage gait, inability to squat fully, atrophy of hands, forearms, lower legs and feet, hand deformity, absent or reduced tendon reflexes, absent Achilles and knee reflexes, sensory ataxia, asymmetric neuropathy, pes cavus (54)	
Compound heterozygotes with L239F (c.715C>T), Pathogenic/Likely pathogenic				
M116T (c.347T>C)	Loop between N- & C-GST domains	Conflicting pathogenicity	AR CMT4C4 Onset in the first decade, frequent falls (8)	M116R GDAP1 does not differ from WT in its ability to protect HT22 cells from glutamate excitotoxicity (36). M116R GDAP1 does not induce mitochondrial fragmentation in COS7 cells (33)
A175P (c.523G>C)	*α*-Loop	Uncertain significance	AR CMT2K Onset in the first decade. At 16 years of age, leg weakness is accompanied by severe weakness of the arms and painful hypesthesia of the hands and feet (24)	
E222K (c.664G>A)	C-term GST	Uncertain significance	AR CMT4A Onset from 1 to 4 years of age. Foot drop, frequent falls, planovalgus foot, calf wasting, Achilles contractures, weak ankle and knee reflexes, preserved upper limb reflexes, sensory neuropathy, flaccid lower-limb paresis, severe limb wasting, pes cavus, distal muscle wasting, hand small muscle involvement, sensory impairment (55)	In neuroblastoma cell line SH-SY5Y with E222K substitution, the level of GDAP1 protein is reduced (51)
**N227D (c.679A>G)**	C-term GST	Uncertain significance	AR CMT4C4 Onset in the first decade. Club feet, gait abnormality (8)	
R273G (c.817C>G)	C-term GST	Uncertain significance	AR CMT4A Onset at 2 years of age. Hand tremor and hoarseness (8)	A comparison of motor neurons derived from pluripotent stem cells from a healthy donor and a patient with the compound heterozygous L239F/R273G mutation showed that these substitutions in GDAP1 reduce mitochondrial Ca^2+^, increase pyruvate dehydrogenase phosphorylation, increase glutamate dehydrogenase activity, and decrease glutamate levels (35). Compared to healthy fibroblasts, fibroblasts from patients with L239F/R273G, GDAP1 have significantly reduced mRNA and protein levels of GDAP1, as well as reduced glutathione levels by 40% and mitochondrial potential by 30% (36)
**R282C (c.844C>T)**	C-term GST	Pathogenic	AR CMT4A Onset at 2–3 years of age. Unsteady gait and toe walking (8, 56, 57)	R282 is in contact to W238 which in turn interacts with hexadecanedioic acid. R282 also forms hydrogen bonds with the backbone carbonyl groups of Q236 and P237 in the α6–α7 loop. All these interactions are lost in human R282H GDAP1, which has also reduced melting temperature (58). The *in vitro* studies refer to R282H GDAP1, not R282C GDAP1 whose clinical phenotype in compound heterozygote with L239F GDAP1 is provided
Other compound heterozygotes				
**P111H**/V219G (**c.332C>A**/c.656T>G)	Loop between N- & C GST/ C-term GST	Uncertain significance/Uncertain significance	AR CMTRIA Onset at 1,5–2 years of age (14).	
Q122 K/N297K (c.364C>A/c.891C>G)	C-term GST/HD	Uncertain significance/Uncertain significance	AR CMT2 Onset at 2.5 years of age. Gait difficulties with distal lower limbs weakness, hand wasting and weakness after age 5, almost complete foot-and-hand paralysis, absent deep tendon reflexes, hypoesthesia, and moderate proximal upper and lower-limb weakness, leading to marked gait impairment (59)	
P153L/A247V (c.458C>T/c.740C>T)	*α*-Loop/C-term GST	Pathogenic/likely pathogenic/Uncertain significance	AR CMT2 Onset at 2 years of age, wheelchair dependence at 36 years of age (6)	A247V in human GDAP1 decreases melting point, shifts positions of α3 and α6 helices due to increased volume of V vs. A residue; affects the intraprotein interaction network involving R120 and H123 (58)
Homozygotes				
W67L (c.200G>T)	N-term GST	Conflicting pathogenicity	AR CMT2K Onset at 18 months. Frequent falls, distal weakness, areflexia, *pes cavus* and sensory ataxia, wheelchair dependence at 17 years of age (60)	
P78L (c.233C>T)	N-term GST	Uncertain significance	AR CMT2 Onset from birth to 2 years of age. Foot deformity from moderate to severe, hoarseness in some patients. High frequency of lost ambulance before 18 years of age (13, 61)	
R120W (c.358C>T)	C-term GST	Pathogenic	AD CMT2 Onset from 4 to 5 years of age, cannot run without orthesis at 10–12 years, pes cavus, clawed hands, CMT neuropathy score 10–15 (13)	Human R120W GDAP1 exhibits decreased thermal stability, a shift in α3 by ∼1 Å; perturbed interaction of Cys240; perturbed salt bridges Glu222-Arg226-Glu229. The variant does not induce morphological changes or toxicity in dorsal root ganglion neurons or HEK293T, does not change cellular localization. Expressed in the cells, WT or R120W GDAP1 do not form intersubunit disulfide bond observed in truncated GDAP1 forms studied *in vitro* (20)
R120Q (c.359G>A)	C-term GST	Uncertain significance	AR CMT4A Onset within the first 2 years of life Axonal loss and demyelination; cranial, sensory, and enteric nerves affected (9)	
D149Y (c.445G>T)	*α*-Loop	Pathogenic	AR CMT2 Only aggregated clinical description for all studied GDAP1 mutants is given (12)	
P153L (c.458C>T)	*α*-Loop	Pathogenic/likely pathogenic	AR CMT2 or AR CMT4C4 Onset from 1 to 3.5 years of age, delayed walking, disturbed gait (42). In some patients hoarseness, paralysis of distal muscles, no ankle jerks, wheelchair dependence at 27 years of age (41)	Probably damaging mutation according to PolyPhen (42)
R161H (c.482G>A)	*α*-Loop	Pathogenic/likely pathogenic	AR CMT4A Onset from 2 to 5 years of age, axonal loss and demyelination, pes cavus, steppage gait, weakness and absence of reflexes may extend to upper limbs (56, 62)	Melting point and stability of R161H GDAP1 are lower, while the conformation is more open, compared to wild type. In SH-SY5Y cells with R161H GDAP1, fewer contacts of mitochondria and endoplasmic reticulum are formed, protein–protein interactions of R161H GDAP1 are perturbed, SOCE^a^ impaired, Ca^2+^ in endoplasmic reticulum decreases (47, 58)
P231L (c.692C>T)	C-term GST	Pathogenic/likely pathogenic	AR CMT2K Onset between the first and forth decade. Frequent falls and limitations in mobility, gait and balance disturbances, difficulties in running and jumping, frequent tripping, high-arched feet with muscle atrophy and depressed deep tendon reflexes (7, 43, 63, 64)	
**L239F (c.715C>T)**	C-term GST	Pathogenic/likely pathogenic	AR CMTRIA Onset in the first decade. Hand weakness and atrophy, along with mild sensory disturbances. Severe disability in the third decade. Mixed axonal and demyelinating form. The mutation is recurrent in the European population (48, 65, 66)	In yeast model L239F GDAP1 decreases GDAP1 expression; impairs trans-Golgi structure, impairs maturation and localization of trans-Golgi network integral membrane protein TGN46; decreases mitochondrial fission; affects distribution of ADP-ribosylation and guanine nucleotide-exchange factor Sec7 in trans-Golgi; increases the rate of DNA exit from mitochondria (67)
A247V (c.740C>T)	C-term GST	Uncertain significance	AR CMT2 Onset at 1 year of age. The most frequent variant in Japanese cohort. Gait disturbance, leg weakness, unstable walking (6)	A247V GDAP1 has decreased melting point; increased volume of V vs. A residue shifts positions of α3 and α6 helices; the substitution affects the intraprotein interaction network involving R120 and H123 (58)
H256R (c.767A>G)	C-term GST	Pathogenic/likely pathogenic	AR CMTRIA Onset from 1.5 to 2.0 years of age. Severe neuropathy (14). Prevalent occurrence in China (16)	
G271R (c.811G>A)	C-term GST	Conflicting pathogenicity	AR CMT4A Onset at 9 years of age. Motor regression, frequent falls, and lower-limb joint locking (19)	
G327D (c.980G>A)	TM	Pathogenic/likely pathogenic	AR CMT2C or AR CMTRIA Onset at 2 years of age. Never was able to run, awkward gait, progressive difficulty to walk long distance, foot deformity. Disease phenotype is similar to the truncated GDAP1 variants (18)	In yeast model, G327D GDAP1 is localized in cytoplasm, partly associating with mitochondria without transmembrane integration; mitochondrial fission decreases compared to WT GDAP1 transfection, but no change in mitochondrial morphology compared to control transfection (18)

a SOCE, store-operated calcium entry.

*GDAP1* missense mutations in the ClinVar database (accessed 11 September 2026) with published records of clinical phenotypes and zygosity states are selected, reported relative to the reference *GDAP1* coding and protein sequences (NM_018972.4/NP_061845.2). Germline classification of the considered GDAP1 variants is taken from ClinVar database. In all presented cases, walking difficulties due to distal muscle weakness and atrophy are common initial symptoms. Only the more variable clinical symptoms are shown in the table. AD—autosomal dominant; AR—autosomal recessive. Regions of GDAP1 are named according to Figure 1. N-term—N-terminal; C-term—C-terminal. The residues most vicinal to the GDAP1-bound hexadecanedioic acid are marked in bold. *In vitro* studies with human GDAP1 and its variants (20) are done for soluble GDAP1 *Δ*303–358 lacking TM and most of HD.

Clinical phenotypes of different *GDAP1* mutations have also been aggregated by AR or AD inheritance and compared to infer genotype–phenotype relationship (13–16). AR forms are often referred to as much more severe regarding the onset and progress of disease than AD forms. However, specific cases do not necessarily support this view (13, 17). For instance, the homozygous phenotype of AR CMT2K caused by P231L GDAP1 is not more severe than the heterozygous phenotype of AD CMT2K caused by C240R GDAP1 (Table 1). Conversely, in the homozygous state, the dominant R120W and the recessive L239F substitutions in GDAP1 result in a similar age at onset and comparable disease progression (Table 1). It has also been noted that truncating *GDAP1* mutations, such as nonsense and frameshift mutations, are associated with more severe CMT phenotypes than missense mutations. However, specific cases contradict this assumption, as the missense mutation leading to G327D GDAP1 shows a severe phenotype of AR CMT2C, similar to that of the truncating mutations (18). As a result, the whole-gene-focused approaches are unable to provide a genotype–phenotype relationship, which would be plausible to apply to specific cases on the one hand while simultaneously withstanding an increasing number of disease-inducing genes and mutations on the other hand.

An important aspect overlooked by a purely genetic view is the idea that the relationship between genotype and phenotype is mediated by the gene-encoded protein, which may have multiple functions. Different mutations may affect distinct functions of the same protein to varied extents, thereby giving rise to diverse clinical phenotypes. Appreciation of this viewpoint has been partially addressed by the gene-locus-dependent cоnsiderations (15). Although more specific, this view still considers linear sequences instead of 3D structures of proteins, thus being unable to incorporate current knowledge on the protein structure–function relationships underlying the phenotypic changes.

Recognizing that the encoded protein mediates the genotype–phenotype relationship provides a more informative framework for understanding this relationship. Various phenotypes of different mutations in a single causal gene may depend on the exact position of the truncation or substitution in the protein sequence, as different regions or specific amino acid residues have unequal structural and/or functional roles in a protein. Moreover, in the case of missense mutations, the phenotype is also dependent on the substituting amino acid. For instance, specific amino acid residues substituting R120 of GDAP1 determine either the AD or AR phenotypes (Table 1). Substitution of the polar arginine residue with polar glutamine (R120Q), which likewise contains a terminal –NH₂ group, results in an AR phenotype. By contrast, substitution of the positively charged arginine residue with the nonpolar tryptophan or glycine residues (R120W and R120G) results in an AD phenotype. The substitutions of positively charged arginine residue by hydrophobic tryptophan or glycine residues (R120W, R120G) result in the AD phenotype. Thus, in the heterozygous state, the polar to polar substitution is not clinically obvious (AR phenotype), while polar to hydrophobic substitutions are obvious (AD phenotypes). In the homozygous state, the phenotype dependence on the chemical nature of the substituting amino acid is further highlighted by the clinically different CMT forms: R120W GDAP1 is associated with AD CMT2, showing axonal damage, while R120Q is associated with AR CMT4A, characterized by axonal and demyelinating damage (Table 1).

The truncating and missense mutations of the same gene have an essential difference regarding their effects on gene-encoded protein expression. The truncating mutations usually reduce protein expression already at the RNA level through nonsense-mediated mRNA decay. In contrast, the missense mutations lead to expression of the full-length protein with modified properties. Hence, truncating mutations cause total loss of all protein functions, while missense mutations may specifically impair selected functions, as well as cause gain of function. In fact, some truncated forms may be expressed and stable at the protein level, while missense mutations may destabilize the protein—decreasing its cellular abundance—or cause start loss [missense mutation c. 1A>G in *GDAP1,* causing Met1Val (19)]. However, the fundamental difference between the absence of the protein and the presence of the functionally altered protein should generally be considered when comparing these mutation types. In this regard, knockout or knockdown of the protein can be used to model truncating mutations, whereas the cellular consequences of missense mutations that permit protein synthesis should be studied using cellular expression of specific protein variants. For instance, in the above-mentioned case of the missense mutation leading to G327D GDAP1, the similarity of its biological impact with that of the nonsense/frameshift mutations is due to the absence of membrane interaction of G327D GDAP1, as confirmed in cells expressing this variant (18). The substitution of non-charged glycine residue by the negatively charged aspartate residue in the hydrophobic transmembrane domain (TM) prevents membrane integration of GDAP1. Similarly, C-terminal GDAP1 truncations also prevent protein binding to the mitochondrial membrane. The missense mutations in other parts of GDAP1 should not necessarily impair membrane binding and may therefore have other phenotypes dependent on the GDAP1 functions beyond membrane binding. Indeed, no changes in the intracellular localization have been shown for the R120W and H123R variants of GDAP1 (20). However, their clinical phenotypes (Table 1) indicate that some other biologically important functions of GDAP1 are perturbed in these variants.

The reportedly greater severity of AR than AD *GDAP1*-associated CMT (13–16) cannot be attributed solely to the molecular pathogenicity of GDAP1 amino acid substitutions. The comparison is strongly confounded by a generic—and therefore mechanistically uninformative—gene dosage effect, as one effectively compares the state with one mutant and one wild-type allele to the state with two mutant alleles. The absence of wild-type alleles in AR cases precludes adaptive upregulation of native-protein synthesis, which remains possible in AD heterozygotes retaining one wild-type allele. Remarkably, YY1, which activates *GDAP1* transcription, is crucial for developmental myelination and responds to genotoxic stress (21). Mutant-allele-induced impairments could therefore trigger YY1-dependent upregulation of wild-type *GDAP1* expression in a timely manner in AD cases. Such upregulation may prevent the vicious cycle of irreversible damage accumulation in AR cases, thereby contributing to milder AD phenotypes compared with the early onset and rapid progression of AR CMT.

Apart from the capability to synthesize native GDAP1, the extent of mutation-induced molecular impairment of GDAP1 function(s) is an essential determinant of the severity of the clinical phenotype and disease trajectory. To further link CMT clinical phenotypes to specific GDAP1 functional impairments and cellular consequences of perturbations in these molecular functions, current knowledge on the GDAP structure–function relationships and cellular changes linked to GDAP1 impairments is reviewed in the following sections.

## Molecular structure and functions of *GDAP1*

According to bioinformatic analysis of the sequence similarity, GDAP1 belongs to the glutathione-S-transferase (GST) superfamily. Its members detoxify electrophilic xenobiotics, including drugs, environmental pollutants, and endogenous toxins, by coupling their reactive groups to glutathione (22). The parts of the GDAP1 sequence which are referred to as the N- and C-terminal GST domains (Figure 1A), align with the N-terminal (glutathione-binding) and C-terminal (hydrophobic-ligand-binding) domains of GST (23). Compared with GST, the C-terminal GST domain of GDAP1 has a rather long insertion in the sequence, called *α*-loop (Figure 1A). The *α*-loop cannot be visualized in the crystal structures due to its high conformational mobility, but the modeling shows dependence of its helical structure on the GDAP1 substitutions in this region (20, 24). In Figure 1B, based on the PDB structure of dimeric GDAP1 with the hydrophobic ligand hexadecanedioic acid (PDB ID: 7AIA) (25), the protein is shown with a previously created model of the *α*-loop structure (24) to enable better analysis of the contribution of the loop residues to CMT phenotypes.

**Figure 1:**
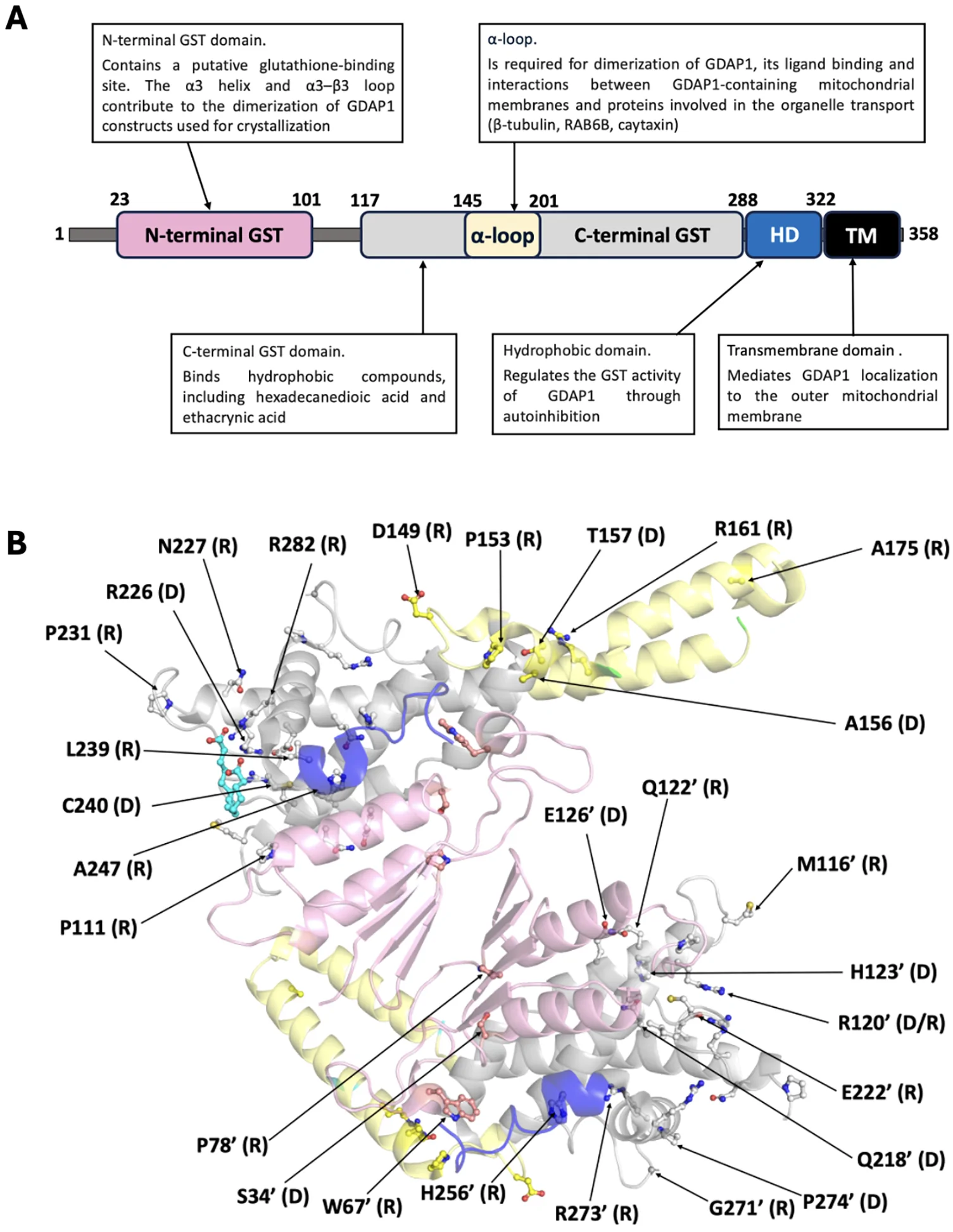
Domain architecture **(A)** and 3D structure **(B)** of GDAP1. The color code for the GDAP1 domains and *α*-loop is shown in **(A)**. In **(B)**, the 3D structure of GDAP1 dimer in cartoon representation and the carbon backbone of explicitly displayed residues affected by substitutions (in ball-and-stick models) use the same color code as in **(A)**. Standard color code for the heteroatoms (red for oxygen, blue for nitrogen, and yellow for sulfur) is used for the displayed amino acid residues. The residues affected by the *α*-loop variants are annotated in the upper subunit. Other substituted residues in GST domains (excluding *α*-loop) are annotated in the different subunits according to their distance to hexadecanedioic acid, measured in the upper subunit. For the residues annotated in the upper subunit the distance is from 3.4 Å (R282) to 14.6 Å (P231). For the residues annotated in the lower subunit, the distance is from 15.1 Å (H256) to 23.4 (Q218) Å. In parentheses, *GDAP1* mutation inheritance types (R—recessive, D—dominant) are shown for the substitutions listed in Table 1. The 3D structure of GDAP1 dimer (PDB ID: 7AIA) includes the *α*-loop regions modeled by AlphaFold and superimposed on the structure using the PyMOL align method (24). The boundaries of the *α*-loop regions that are not visible in the X-ray structure are indicated in blue for the lower subunit and in green for the upper subunit.

The two GST domains of GDAP1 face the cytosol, followed by the two additional hydrophobic domains specific to GDAP1 (Figure 1A). The C-terminal TM localizes GDAP1 to the outer mitochondrial membrane, while the preceding hydrophobic domain (HD) regulates the GST activity of a soluble GDAP1 construct through a conformational switch involved with autoinhibition (26). Although the GDAP1 dimer is formed regardless of the presence of HD, HD renders the GST activity of this construct undetectable. Based on these data, the mechanism of HD-mediated autoinhibition of GDAP1 catalysis, potentially sensing membrane curvature through membrane positioning of amphipathic helices of HD, has been suggested, linking the GST and mitochondrial fission activities of GDAP1 (26). Figure 1A summarizes the significance of GDAP1 structural parts for its specific molecular and biological functions.

A dimeric state of GDAP1 possessing GST activity has been shown for GDAP1 produced without HD and TM in the eukaryotic expression system, but this catalytically active form could not be crystallized (26). The crystallized dimeric form of GDAP1 lacking the C-terminal part (Figure 1B) is also inherent in six studied variants of GDAP1 whose 3D structures are currently deposited in the Protein Data Bank. In this regard, the crystallographic data agree with biochemical evidence on the dimeric structure of soluble and catalytically active GDAP1 expressed in the eukaryotic system (26). However, the crystallized dimeric structures of GDAP1 expressed in bacterial system have an intersubunit disulfide bond between C88 residues of the N-terminal GST domains of GDAP1 subunits, which is absent from native cellular GDAP1 (20, 25). Hence, the difference between the dimerization interfaces of crystallized GDAP1 constructs and GST, as well as the lack of GST activity in the dimeric GDAP1 configuration reported in these studies (20, 25), may be due to the non-native disulfide bond formed at the subunit interface of GDAP1 expressed in the bacterial system. In view of different systems controlling the formation of protein disulfide bonds in pro- and eukaryotes (27), the eukaryotic expression of active GDAP1 probably prevents formation of the non-native disulfide bond between monomers of GDAP1, which occurs upon the protein expression in bacterial system. This folding difference may explain the presence of GST activity only in GDAP1 obtained upon eukaryotic expression (26). Together with another artifact of GDAP1 studies, showing monomeric structure of the GDAP1 construct that misses not only the C-terminal part but also *α*-loop essential for dimerization (PDB ID 6UIH) (28), the current data justify further efforts to crystallize GDAP1 in its native membrane-bound state.

Despite these limitations, the available structural information provides important insights. According to a minimal model assuming equal abundance of wild-type and variant monomers, comparable dimerization affinities, and random association, GDAP1 dimerization in affected heterozygotes would yield 25% wild-type/wild-type, 25% variant/variant, and 50% wild-type/variant dimers. The actual distribution may differ because both the expression and oligomerization of GDAP1are dynamic and context-dependent, potentially affected by unequal expression of the two alleles, differences in the stability and dimerization affinities of native and variant subunits, and other factors. For instance, adaptive upregulation of GDAP1 expression by YY1 (21) and/or elimination of the variant subunits due to their instability could increase the relative abundance of wild-type protein and, consequently, the proportion of native GDAP1 dimers. Conversely, the relative proportion of wild-type GDAP1 homodimers could theoretically be reduced if wild-type/variant dimerization were favored over wild-type/wild-type dimerization, for example, through preferential binding of variant subunits to wild-type subunits. However, no data supporting such a mechanism are available, whereas reduced stability has been demonstrated for some missense GDAP1 variants (Table 1). Irrespective of the actual dimer distribution, a fundamental difference remains in the capacity to express native GDAP1: AD heterozygotes can synthesize it, whereas AR homozygotes and compound heterozygotes cannot. Analysis of the data presented in Table 1 shows that this difference corresponds to exclusively axonal damage in heterozygous AD cases, whereas AR cases with homozygous or compound heterozygous GDAP1 variants often demonstrate additional demyelination. The involvement of GDAP1 transcriptional regulator YY1 in both developmental myelination (29) and upregulation of GDAP1 expression (21) offers a mechanistic explanation for different disease trajectories in these AD and AR cases.

Another phenotype-related implication of structural studies is the half-of-the-sites reactivity of GDAP1, pointing to functional coupling (cooperativity) between the dimer subunits. This is indicated by no simultaneous binding of hexadecanedioic acid in the GDAP1 dimer subunits with equal sequences (Figure 1B), and the different conformations of these subunits in the crystallized dimeric structures of GDAP1 (20, 24). If the binding sites act in a coupled way, some pathogenic substitutions in only one subunit may compromise the function of the second, wild-type subunit of GDAP1, thereby impairing function of both subunits in the wild-type/variant heterodimer.

Structural data also allow one an assessment of the distribution and potential functional significance of CMT-associated substitutions of amino acid residues in 3D structure of GDAP lacking HD and TM (Figure 1B). Together with the data in Table 1, analysis of Figure 1B indicates that most of the characterized clinical phenotypes of GDAP1-associated CMT refer to the protein variants in C-terminal GST domain including *α*-loop. Based on ClinVar data, both overall and pathogenic or likely pathogenic missense-variant densities, defined as the numbers of variants per 100 amino acid residues, are lower in the N-terminal GST domain and transmembrane domain (TM, Figure 1) than in the C-terminal GST domain and hydrophobic domain (HD, Figure 1): Overall variant densities are 67 and 65 in the former domains, respectively, compared with 82 and 79 in the latter domains, respectively, whereas the corresponding pathogenic or likely pathogenic variant densities are 5 and 3 vs. 14 and 9. This distribution allows one to hypothesize that the N-terminal GST domain and TM are less tolerant of amino acid substitutions than the C-terminal GST domain and HD. The associated reduction in variants compatible with postnatal survival in the former compared with the latter domains could lead to underrepresentation of variants in the N-terminal GST domain and TM in clinical observations (Table 1).

Based on their functional significance, the substituted amino acid residues in the C-terminal GST domain of GDAP1 (Figure 1B) can be classified into three groups. The first group comprises the residues at the base (D149, P153, A156, T157, and R161) and tip (A175) of *α*-loop. Substitutions of these residues may affect the overall conformation and hinge motion of the loop, potentially involved in closing the active site of GDAP1 (20, 24). The substitutions A156G and T157P at the start of the *α*-loop helical structure cause AD phenotypes, in accordance with their critical involvement with the functionally important hinge motion (20). In particular, in T157P GDAP1, the *α*-loop is destabilized and changes its dynamics (30).

The second group includes the residues within 15 Å to the GDAP1-bound hydrophobic ligand. In Figure 1B, they are annotated in the gray part of the C-terminal GST of the upper subunit. Substitutions in R226 and C240 result in AD phenotypes (Table 1), suggesting essential roles of these residues within the binding site. The third group involves the residues potentially affecting those of the second group through the intramolecular interaction network (20). These residues in the gray part of the C-terminal GST domain, annotated in the lower subunit in Figure 1B, are >15 Å away from the hydrophobic ligand. In particular, in native GDAP1, R120 interacts with the binding site residues R226 and C240. The interaction is perturbed in R120W GDAP1, potentially affecting the binding of the hydrophobic ligand. The substitution H123R also affects the intramolecular interaction network in GDAP1, particularly the salt bridges with participation of R226 close to the hydrophobic ligand (20). As all these substitutions are associated with the AD phenotypes of CMT, impairment of hydrophobic ligand binding, potentially heightened by half-of-the-sites reactivity of subunits in the GDAP1 dimer, appears to be among the critical determinants of the disease mechanism.

## Biological consequences of *GDAP1* impairments

Membrane localization of GDAP1 is important for the formation of mitochondrial contacts with endoplasmic reticulum, lysosomes, peroxisomes (31), and plasma membrane, with the latter contacts mediating store-operated calcium entry (SOCE) (32). Accordingly, GDAP1 is considered to be a regulator of mitochondrial network in cells (33). In different cellular models, manipulations of GDAP1 expression affect mitochondrial morphology, calcium homeostasis, membrane potential, and metabolism (Table 1). However, GDAP1 silencing or pathogenic mutations do not necessarily change mitochondrial morphology. For instance, fibroblasts of a patient with AD CMT2K due to H123R substitution in GDAP1, or root dorsal ganglion neurons transfected by GDAP1 with R120W or H123R substitutions, do not show morphological changes (20). In primary skeletal muscle cells with GDAP1 silenced via siRNA transfections, mitochondrial morphology is not changed, but mitochondrial protein abundance increases, while palmitate oxidation and non-mitochondrial respiration decrease (34). In CMT-patient-derived fibroblasts and *GDAP1*^−/−^ mice, impaired contacts of mitochondria and lysosomes propagate to defective interactions of mitochondria with peroxisomes, affecting biogenesis and morphology of peroxisomes and altering lipid metabolism (31). Inconsistent сhanges in mitochondrial morphology across GDAP1 dysfunctions argue against mitochondrial morphological alterations as the primary pathogenic mechanism. This notion is further supported by similarity and relatively mild phenotypes of AD CMT2K upon R120W, H123R, and A156G substitutions, with only the latter one inducing mitochondrial fragmentation in HeLa cells (Table 1).

On the other hand, GDAP1 is highly expressed in neurons where its CMT-associated dysfunction strongly changes key enzymes of mitochondrial metabolism (35). The link between GDAP1 and cellular redox state (31) is independently supported by positive correlations of GDAP expression and cellular glutathione levels (36, 37). Interestingly, in skeletal muscles, GDAP1 expression is associated with regulation of circadian genes, is decreased by AMPK or exercise, but increased in the muscles of patients with type 2 diabetes (34). Although molecular mechanisms of all these associations remain unknown, involvement of GDAP1 in insulin-signaling and mitochondria-dependent neuromuscular degeneration is also shown in the *Drosophila* model (38). One may therefore conclude that the link between GDAP1 variants and their effects on organellar morphology is mediated by cell-specific circuits involved in metabolic regulation. This is further supported by the common increase in phosphorylation of pyruvate dehydrogenase in neuronal cells expressing CMT-associated variants of either *GDAP1* (35) or *PDK3*, encoding pyruvate dehydrogenase kinase 3 (39), and the beneficial effect of thiamine—the precursor of the pyruvate dehydrogenase coenzyme—in GDAP1-associated CMT (24).

The complexity of neurological function and interindividual variation in the expression of non-mutated genes may underlie the known, often intrafamilial, variability in the clinical manifestations of a specific CMT type. This possibility is illustrated by attenuation of the consequences of GDAP1 knockdown in a human neuronal cell line through expression of a mitophagy-mediating protein BNIP3, while simultaneous knockdowns of both genes are more detrimental than knockdown of GDAP1 alone (40). Such interactions may explain how the same causal mutation may be associated with clinically different CMT types, as occurs with P153L GDAP1 (Table 1) leading to AR CMT2 or AR CMT4C4 (41, 42). Transcriptional regulation of *GDAP1*, including epigenetic regulation (21), represents yet another factor potentially contributing to variable clinical manifestations of a specific GDAP1 variant.

A better understanding of the biological significance of molecular functions of GDAP1, its protein interaction network, and transcriptional regulation will allow for better diagnostics, prognosis, and treatment of CMT.

## Conclusions

The diverse clinical phenotypes associated with various mutations within a single causal gene are mediated by the perturbed expression of the encoded protein resulting from truncating mutations or by the distinct properties of protein variants resulting from missense mutations. Considering how mutation-induced protein variants affect specific molecular functions of GDAP1 provides greater insight into the molecular mechanisms underlying clinical phenotypes than correlating these phenotypes with AR or AD inheritance, mutation type (truncating vs. missense), or the cellular consequences of the mutations. However, current implementation of the advanced understanding of the genotype–phenotype relationship is limited by the lack of experimental characterization of mammalian proteins in their native state. In this regard, GDAP1 is representative of many other proteins whose molecular functions may be predicted by bioinformatics analysis, but remain unconfirmed or insufficiently characterized *in vitro* at the protein level. In addition to genetic defects causing decreased GDAP1 translation, knowledge of specific amino acid residues essential for diverse molecular functions of GDAP1 would enable patient stratification according to the primary molecular dysfunction, such as impaired catalytic activity or membrane localization and altered binding of low- and high-molecular-mass ligands. Identification of the primary molecular defect caused by a specific variant could also improve its functional interpretation and guide the selection of assays appropriate for quantifying impairment of the affected GDAP1 function. In turn, this knowledge could inform prognosis and guide mechanism-based therapeutic strategies that target the primary defect as specifically as possible. Our current understanding of the structure–function relationships in GDAP1 and their impact on the associated CMT phenotypes is challenged, on the one hand, by the variety of cell types and assays employed to characterize these relationships and, on the other hand, by the lack of quantitative data both in these studies and in clinical descriptions. Better standardization and quantification of clinical phenotypes and disease progression, along with a better understanding of molecular functions and structure–function relationships in the proteins encoded by the disease-associated genes, are required to achieve the goals of personalized medicine.
